# Protective Effect of Galectin-1 during* Histoplasma capsulatum* Infection Is Associated with Prostaglandin E_2_ and Nitric Oxide Modulation

**DOI:** 10.1155/2016/5813794

**Published:** 2016-09-06

**Authors:** Lílian Cataldi Rodrigues, Adriana Secatto, Carlos A. Sorgi, Naiara N. Dejani, Alexandra I. Medeiros, Morgana Kelly Borges Prado, Simone Gusmão Ramos, Richard D. Cummings, Sean R. Stowell, Lúcia Helena Faccioli, Marcelo Dias-Baruffi

**Affiliations:** ^1^Departamento de Análises Clínicas, Toxicológicas e Bromatológicas, Faculdade de Ciências Farmacêuticas de Ribeirão Preto, Universidade de São Paulo (USP), 14040-903 Ribeirão Preto, SP, Brazil; ^2^Departamento de Ciências Biológicas, Faculdade de Ciências Farmacêuticas, Universidade Estadual Paulista (UNESP), 14801-902 Araraquara, SP, Brazil; ^3^Departamento de Patologia, Faculdade de Medicina de Ribeirão Preto, Universidade de São Paulo (USP), 14049-900 Ribeirão Preto, SP, Brazil; ^4^Department of Surgery, Beth Israel Deaconess Medical Center and Harvard Medical School, Boston, MA 02115, USA; ^5^Department of Pathology, Emory University School of Medicine, Atlanta, GA 30322, USA

## Abstract

*Histoplasma capsulatum* is a dimorphic fungus that develops a yeast-like morphology in host's tissue, responsible for the pulmonary disease histoplasmosis. The recent increase in the incidence of histoplasmosis in immunocompromised patients highlights the need of understanding immunological controls of fungal infections. Here, we describe our discovery of the role of endogenous galectin-1 (Gal-1) in the immune pathophysiology of experimental histoplasmosis. All infected wild-type (WT) mice survived while only 1/3 of Lgals1^−/−^ mice genetically deficient in Gal-1 survived 30 days after infection. Although infected Lgals1^−/−^ mice had increased proinflammatory cytokines, nitric oxide (NO), and elevations in neutrophil pulmonary infiltration, they presented higher fungal load in lungs and spleen. Infected lung and infected macrophages from Lgals1^−/−^ mice exhibited elevated levels of prostaglandin E_2_ (PGE_2_, a prostanoid regulator of macrophage activation) and prostaglandin E synthase 2 (*Ptgs2*) mRNA. Gal-1 did not bind to cell surface of yeast phase of* H. capsulatum*,* in vitro*, suggesting that Gal-1 contributed to phagocytes response to infection rather than directly killing the yeast. The data provides the first demonstration of endogenous Gal-1 in the protective immune response against* H. capsulatum *associated with NO and PGE_2_ as an important lipid mediator in the pathogenesis of histoplasmosis.

## 1. Introduction

Histoplasmosis is a worldwide known disease caused by the fungus* Histoplasma capsulatum*. The real geographic distribution of this mycosis could be more widespread than what was previously thought [1, 2]. The incidence of this fungal disease is higher in the Mid- and Southeast USA, Latin America, China, and other world areas [2]. Additionally, asymptomatic cases are escalating and are reported to predominately affect immunocompromised individuals as an acute pulmonary infection similar to mild flu-like symptoms [1, 3, 4]. Likewise, the most severe symptomatic form of the disease, referred to as disseminated histoplasmosis, develops most commonly in immunosuppressed patients. However, unlike the mild form, disseminated histoplasmosis can lead to death [4]. Although antifungal therapies have been used against the fungus, there are no current alternative therapies to treat or protect against* H. capsulatum *infection.

*H. capsulatum* is a dimorphic, facultative, intracellular pathogen found as a yeast phase when in host tissue [5]. In the early stages of infection, the fungus is phagocytosed by resident alveolar macrophages, dendritic cells, and neutrophils [6]. Once phagocytosed, the fungus survives in the phagosome and consequently transforms into a yeast. In immunocompromised individuals or when left untreated, the reservoir phagocytes can travel to lymphatic tissue and spread infection. However, induction of a strong cellular immune response can contain or clear the infected phagocytes, therefore preventing the spread of the infection. An effective host defense to* H. capsulatum* infection is dependent on adequate activation of T cells and phagocytes [6, 7]. Appropriately, the balance between the Th1 and Th2 response is fundamental for solving* H. capsulatum* infection [6, 7], with Th1 proinflammatory cytokines IFN-*γ* (interferon-*γ*), interleukin-12 (IL-12), TNF-*α* (tumor necrosis factor-*α*), and GM-CSF (granulocyte macrophage colony-stimulating) being essential to elicit macrophage activation and clearance of* H. capsulatum*. In addition, a balanced production of lipid mediators, such as prostaglandin E_2_ (PGE_2_) and leukotriene B_4_ (LTB_4_), is critical for the host defense in histoplasmosis, since high levels of PGE_2_ and low levels of LTB_4_ impair the yeast clearance and increase the severity of this fungal disease [8, 9]. Nitric oxide also participates in the host defense against* H. capsulatum* [10, 11]; however, overproduction of this mediator increases the susceptibility of the host to yeast infection [8, 12].

In addition to cytokines and lipid mediators, a member of the galectin family, known as galectin-3 (Gal-3), has been suggested to be involved in the immune response against* H. capsulatum* infection [13]. Galectins belong to an endogenous lectin family that recognizes glycans present in microorganisms and participates in the pathophysiology of inflammatory responses, infectious diseases, autoimmunity, and cancer [14–18].

Interestingly, galectin-1 (Gal-1) has been shown to participate in an innate and adaptive immune response to different models of experimental infections such as in* Trypanosoma cruzi *(*T. cruzi*) [19], situation in which a dual role for this lectin was described. These authors showed that, in a low concentration, Gal-1 was able to decrease proinflammatory interleukin-12 (IL-12) and nitric oxide (NO), while in a high concentration, it has induced infected macrophage apoptosis. Gal-1 was also found to promote* Human Immunodeficiency Virus-1* (HIV-1) infectivity [20]; in dengue virus infection, it could cause an inhibitory effect on virus replication [21]. Thus, several Gal-1 exogenous properties have been related to CRD binding to cell surface receptors, modulating immune cell functions, migration, differentiation, activation, and cell survival [22–27]. Nevertheless, the interactions of this lectin with the intracellular ligands can also occur independently to carbohydrates [28, 29].

Although Gal-1 can participate in various pathophysiological processes, there is little information about the role of Gal-1 in fungal infections. Therefore, the present study evaluated the biological impact of the absence of Gal-1 on a murine model of histoplasmosis. While mice genetically deficient in Gal-3 (Lgals3^−/−^) were able to clear* H. capsulatum* infection more efficiently than wild-type (WT) mice [13], it was reported for the first time that Gal-1 (Lgals1^−/−^) mice are more susceptible to* H. capsulatum* infection compared to WT group. This unique immune phenotype suppresses the host response against the fungus and is followed by high levels of neutrophil infiltration and proinflammatory cytokines in the lungs which causes a strong anti-inflammatory response with high levels of PGE_2_ and NO. These findings indicate a novel contribution of endogenous Gal-1 to the development of a protective immune response to* H. capsulatum.*

## 2. Results

### 2.1. Lgals1^−/−^-Infected Mice Fail to Control* H. capsulatum* Infection

**Figure 1:**
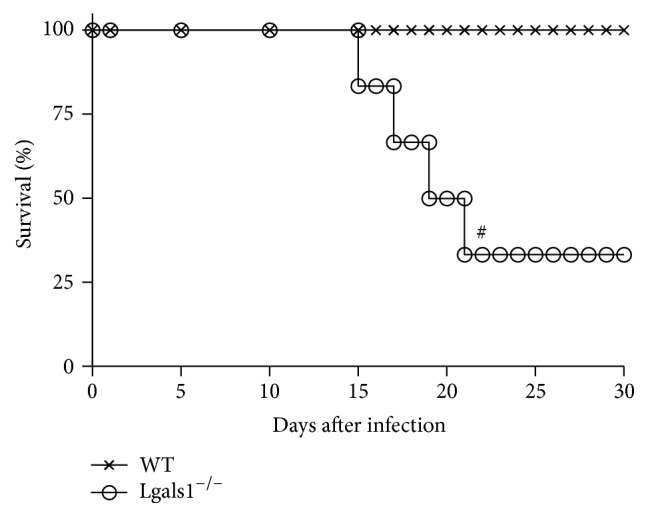
Gal-1 knockout mice are sensitive to* H. capsulatum* infection. WT and Lgals1^−/−^ mice were infected i.t. with 5 × 10^5^ viable* H. capsulatum* yeast (sublethal inoculum) and survival was monitored for 30 days. Control mice (uninfected) received i.t. 100 *μ*L PBS (data not shown). Data are representative of one of the two experiments performed independently (*n* = 10 per group) and Mantel-Cox log-rank (*χ* ^2^ “chi-squared”) was used. ^#^ *p* < 0.05 WT versus Lgals1^−/−^, both infected.

WT and Lgals1^−/−^ mice were injected with 5 × 10^5^ *H. capsulatum *yeasts cells directly into the lungs and survival was monitored up to 30 days. 14 days after infection, Lgals1^−/−^-infected mice began to die. 33% of Lgals1^−/−^-infected mice survived 30 days after infection, whereas 100% of the infected WT mice survived over that period (Figure 1).

### 2.2. Lgals1^−/−^-Infected Mice Have a Higher Yeast Load and Infiltration of Neutrophils in the Lung

**Figure 2:**
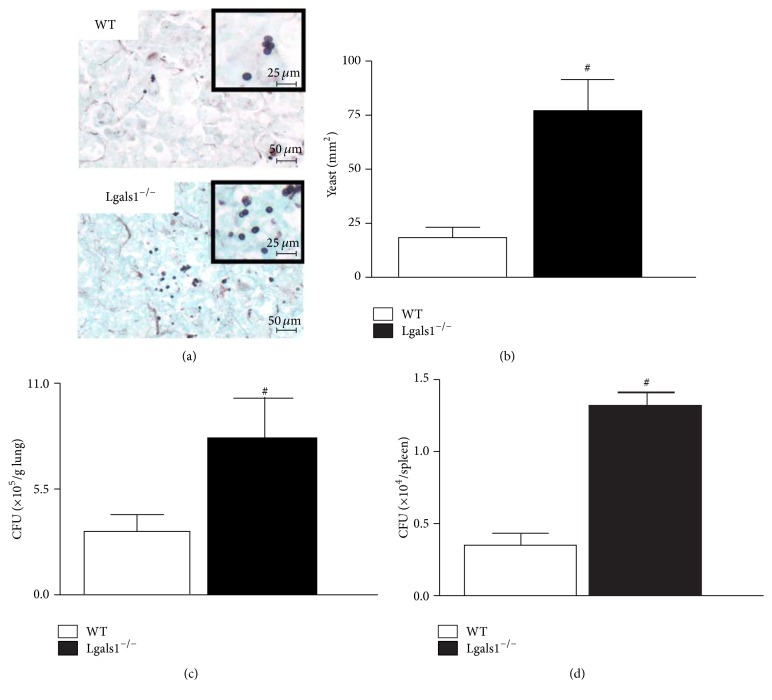
Lgals1^−/−^ mice have a higher yeast burden in the lung and spleen day 15 after infection. On day 15 after infection with* H. capsulatum* (5 × 10^5^ viable yeasts), animals were sacrificed and tissue samples were harvested. (a) Lung sections (5 *μ*m) from WT and Lgals1^−/−^ mice were stained with silver (Grocott's methanamine silver (GMS); bar: 50 *μ*m; insert bar: 25 *μ*m) and (b) yeast cells were quantified (yeast/mm^2^ lung) using magnifications ×400. Fungal burden was quantified from tissue homogenates and expressed as the number of colony-forming units (CFU) per gram of tissue CFU/g in lung (c) and CFU per spleen (d). Data are representative of one of the two experiments performed independently (*n* = 10 per group). Values are mean ± SEM. ^#^ *p* < 0.05 WT versus Lgals1^−/−^, both infected.

**Figure 3:**
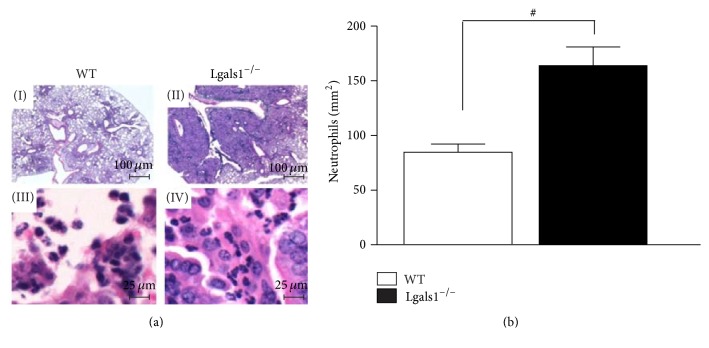
Lgals1^−/−^ mice have increased neutrophil infiltration in the lung parenchyma.* H. capsulatum*-infected mice were euthanized on day 15 after infection and lung sections (5 *μ*m) were embedded in paraffin blocks. Lung sections from WT +* H. capsulatum *(I, bar: 100 *μ*m; III, bar: 25 *μ*m) and Lgals1^−/−^ +* H. capsulatum* (II, bar: 100 *μ*m; IV, bar: 25 *μ*m) were stained with hematoxylin (a) and neutrophils were quantified (neutrophils/mm^2^) using magnifications ×400 (b). Data are representative of one of the two experiments performed independently (*n* = 10 per group). Values are mean ± SEM. ^#^ *p* < 0.05 WT versus Lgals1^−/−^, both infected.

To determine if the high mortality rate in Lgals1^−/−^ mice is correlated with impaired fungal clearance, the* H. capsulatum* load was quantified in the lung and spleen. Considering that Lgals1^−/−^ mice began to die day 14 after infection and that day 15 after infection is a critical point on the evolution of experimental histoplasmosis using mutant mice and a sublethal fungus dose [9], on day 15 after infection, lung parenchymal histopathological analysis and quantification of fungal burden in the lung and spleen were performed. Lgals1^−/−^-infected mice presented a higher number of yeast cells in pulmonary parenchyma (Figures 2(a) and 2(b)) and higher fungal load in lung (Figure 2(c)) and spleen (Figure 2(d)). Even though infected Lgals1^−/−^ mice presented a higher fungal burden in the lung, an increased neutrophil influx was detected in their pulmonary tissue (Figures 3(a) and 3(b)). It is known that an efficient immune response against* H. capsulatum* is associated with fungicidal/fungistatic effects of pulmonary infiltrated phagocytes [6, 30]. Thus, these findings suggest that endogenous Gal-1 is required to develop a protective immune response against* H. capsulatum *and that Gal-1 could be associated with the control of fungal replication as an efficient anti*-H. capsulatum *activity along with effectors functions and regulation of tissue accumulation of neutrophils.

### 2.3. Lgals1^−/−^-Infected Mice Show Increased Proinflammatory Cytokines in the Lung

**Figure 4:**
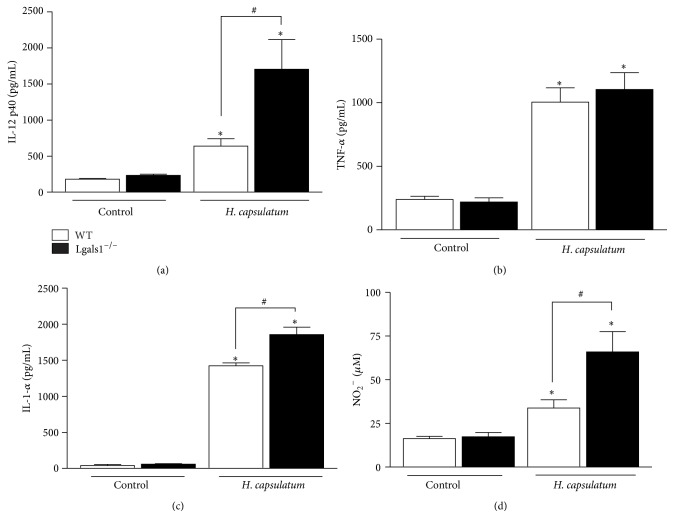
Lgals1^−/−^ mice exhibit increased inflammatory response day 15 after infection with* H. capsulatum*. Cytokines IL-12p40 (a), TNF-*α* (b), IL-1-*α* (c), and NO_2_^−^ (d) were quantified from homogenized lungs on day 15 after infection with* H. capsulatum*. Cytokines levels (pg/mL) were determined in the supernatants by ELISA and NO_2_^−^ (*μ*M) by using a Griess reaction. Data are representative of one of the two experiments performed independently (*n* = 10 per group). Values are mean ± SEM. ^*∗*^ *p* < 0.05 infected mice versus control (uninfected), ^#^ *p* < 0.05 WT versus Lgals1^−/−^, both infected.

It is well known that increased expression of inflammatory cytokines, including IL-12, IFN-*γ,* and TNF-*α*, is critical for the immune-protective response in* H. capsulatum*-infected mice [31–34]. Thus, to analyze the pattern of inflammatory cytokines in WT and Lgals1^−/−^ mice day 15 after* H. capsulatum* infection, the levels of IL-12p40, TNF-*α*, IL-1*α*, IL-10, IL-4, and IL-6 in the pulmonary homogenates were measured. There were higher levels of IL-12p40 (Figure 4(a)) and IL-1*α* (Figure 4(c)) and similar concentrations of TNF-*α* (Figure 4(b)) in homogenized lungs of Lgals1^−/−^-infected mice, when compared to WT infected mice. Furthermore, no statistically significant differences in TNF-*α* (Figure 4(b)) were observed, and IL-10, IL-4, and IL-6 (data not shown) were not produced in detectable levels.

### 2.4. Lgals1^−/−^-Infected Mice Demonstrate Prostaglandin E_2_ and Nitric Oxide Overproduction

**Figure 5:**
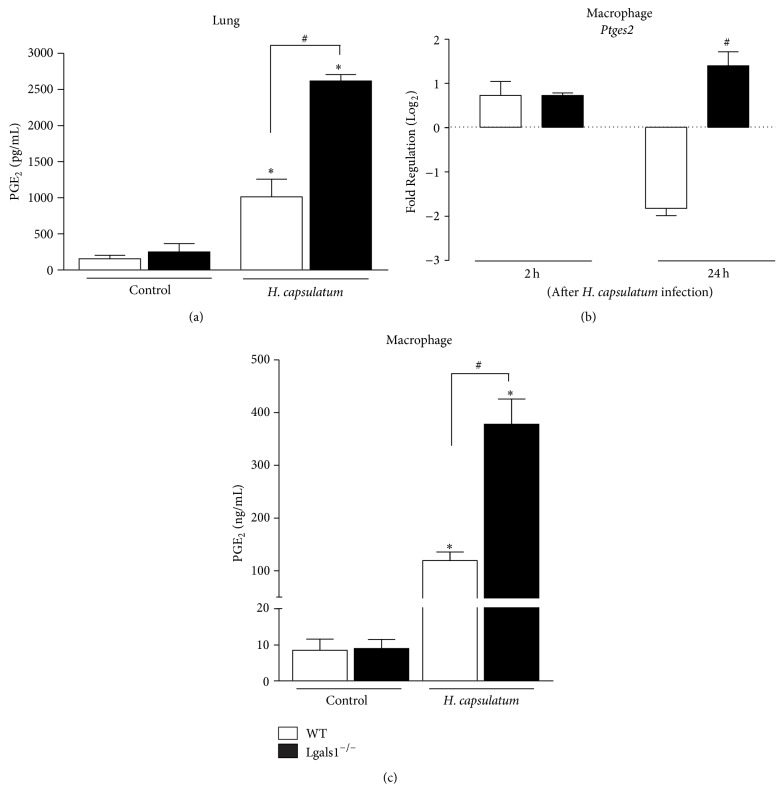
Absence of endogenous Gal-1 increases prostaglandin PGE_2_ production and Ptges2 expression in peritoneal macrophages. (a)* In vivo* prostaglandin E_2_ was quantified in supernatants from homogenized lungs on day 15 after infection with* H. capsulatum* (5 × 10^5^ yeasts/mice) by ELISA. (b) 5 × 10^5^ peritoneal macrophages were incubated* in vitro* with* H. capsulatum* (MOI 1 : 1) during 2 and 24 hours and mRNA levels for Ptges2 were quantified and plotted as Fold Regulation by Log_2_. In addition, PGE_2_ was assessed* in vitro* in the supernatants by ELISA 24 hours after infection (c).* In vivo* data are representative of one of the two experiments performed independently (*n* = 10 per group). Values are mean ± SEM. ^*∗*^ *p* < 0.05 infected mice versus control (uninfected), ^#^ *p* < 0.05 WT versus Lgals1^−/−^, both infected.

Based on the aforementioned results, it was also analyzed whether inflammatory mediators, such as NO and PGE_2_, are associated with increased levels of proinflammatory cytokines and consequently immunosuppression in the absence of endogenous Gal-1 in experimental histoplasmosis. It has been reported that the inhibition of COX-2 improves the host defense against* H. capsulatum* [8]. Therefore, PGE_2_ was quantified from homogenized lungs derived from infected WT and Lgals1^−/−^ mice on day 15 after* H. capsulatum* infection. The lungs of infected Lgals1^−/−^ mice exhibited higher levels of PGE_2_ (Figure 5(a)) when compared to infected WT mice. Thus, consistent with other published results [8, 35], these findings suggest that higher levels of PGE_2_ may contribute to susceptibility of infected Lgals1^−/−^ mice. Interestingly, not only PGE_2_ but also NO levels in the lung of this group were increased when compared to WT (Figure 4(d)).

### 2.5. Uninfected Lgals1^−/−^ Macrophages Express High Levels of Prostaglandin E Synthase 2 after Fungal Infection

The immune response against* H. capsulatum* is mediated by Th1 cells, which requires macrophages activation [6, 7]. The pathogenic yeast fungus replicates inside these cells and results in metabolites of arachidonic acids production, such as prostaglandins and leukotrienes [35]. To assess the role of endogenous Gal-1 in PGE_2_ production, prostaglandin E synthase 2 (Ptges2) mRNA expression in peritoneal macrophages from Lgals1^−/−^ and WT mice infected or not with* H. capsulatum in vitro* was evaluated. Interestingly, 24 hours after* H. capsulatum* infection, Lgals1^−/−^ macrophages had increased Ptges2 mRNA expression when compared to infected WT macrophages. In addition, higher levels of PGE_2_ were detected in the supernatants 24 hours after the infection of Lgals1^−/−^ macrophages when compared to WT macrophages (Figure 5(c)). Thus, the* in vitro* results correlate with overproduction of prostaglandins* in vivo* (Figure 5(a)).

### 2.6. Galectin-1 Does Not Bind to and Kill the Yeast Form of* H. capsulatum*

**Figure 6:**
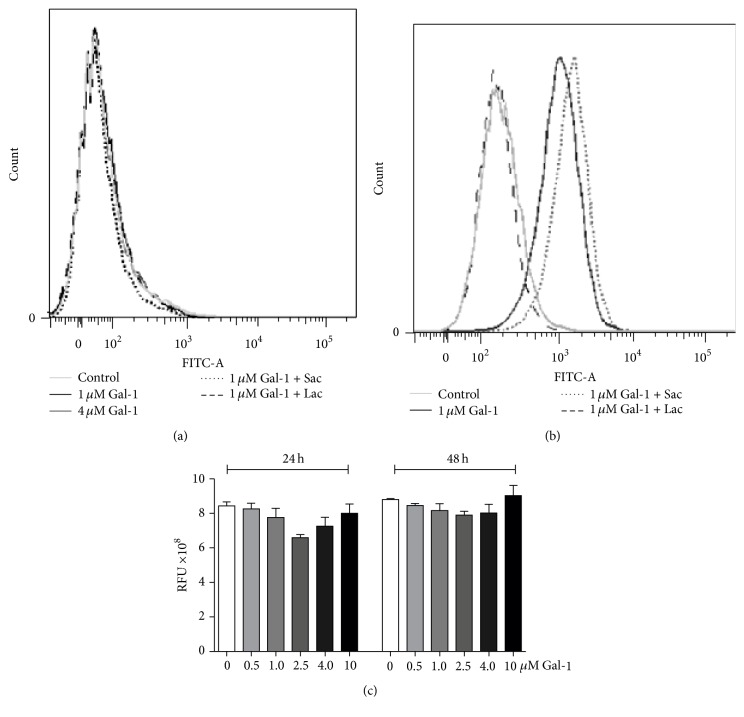
Gal-1 does not bind and kill the yeast form of* H. capsulatum.* (a) Yeasts were incubated for 1 hour at 4°C with 1.0 *μ*M and 4.0 *μ*M biotinylated-hrGal-1, in the presence or absence of 20 mM lactose (Gal-1 inhibitor) or sucrose (control, noninhibitor). After that, yeasts were incubated with streptavidin-FITC and labeled cells were acquired on a FACS Canto (Becton Dickinson, Mountain View, CA, USA) and analyzed in the DIVA software (Becton Dickinson). (b) As a control, HL-60 cells (1 × 10^6^) were incubated with 1 *μ*M biotinylated-hrGal-1 for 1 hour at 4°C, in presence or absence of 20 mM lactose or sucrose. (c) Several hrGal-1 concentrations (0.5, 1.0, 2.5, 4.0, and 10 *μ*M) were incubated with 1 × 10^6^ *H. capsulatum* cells during 24 and 48 h. After each time, relative fluorescent units (RFU) (560–590 nm) were measured and represent yeast cells metabolically active through the dye resazurin reagent. Data are representative of two independent experiments and expressed as the mean ± SEM.

Recently, it was reported that galectins can bind glycans not only on the host cell surface, but also on molecules on pathogens, which has been found to result in pathogen killing and modulation of immune responses against bacterial infections [36, 37]. To assess the binding capacity of Gal-1 on* H. capsulatum* surface, biotinylated-human recombinant Gal-1 (hrGal-1 : 1 *μ*M and 4 *μ*M) was incubated with the yeast form of* H. capsulatum*. Gal-1 did not bind to the yeast form of this fungus (Figure 6(a)) although the hrGal-1 was active, since it did bind to glycans on HL-60 cells (Figure 6(b)). As expected, different concentrations of hrGal-1 (0.5, 1.0, 2.5, 4.0, and 10.0 *μ*M) did not alter the viability of* H. capsulatum* after 24 and 48 h of* in vitro* incubation (Figure 6(c)). This result suggests that the binding effect can be related to killing activity as Stowell et al. [36] described for* E. coli* strains in the presence of Gal-4 and Gal-8. Thus, the yeast form of* H. capsulatum* seems not to express ligands for Gal-1 and indicates that the protective mechanistic effects of Gal-1 to* H. capsulatum* infection do not involve Gal-1 binding to the yeast.

## 3. Discussion

Galectins have been described as regulators of immune response in models of inflammatory and infectious diseases and host pathogen recognition [14, 25, 27, 36, 38–41]. Gal-1 and Gal-3 are the best studied members of the galectin family and the expression of these proteins is increased or decreased in distinct cell types following infections caused by different pathogens [42, 43]. Previous reports demonstrate that Gal-3 participates in yeast infections [13, 39, 44]; however, the role of Gal-1 in fungal diseases has not yet been explored. Although the expression of Gal-3 in dendritic cells is not upregulated in WT mice infected with* H. capsulatum*, mice genetically deficient in Gal-3 clear this fungal infection more efficiently than WT mice [13], showing that high Gal-3 expression in WT mice is not required for the participation in the immune response against* H. capsulatum* and may actually contribute to pathogenesis [13].

Unexpectedly, Gal-3 knockout mice are more susceptible to* Candida albicans* infection than WT mice and the susceptibility is associated with high fungal burden in the brain. Additionally, Gal-3, but not Gal-1, can induce yeast cell death upon binding to *β*-1,2-linked oligomannosides on the surface of pathogenic fungus* Candida albicans* [44]. Thus, Gal-3 and Gal-1 appear to be differentially involved in host defense mechanisms against fungal infections, and this feature may arise from the specific pathogen. In disseminated candidiasis model, the absence of Gal-3 is responsible for increased susceptibility [39]. In the present study, in contrast to Gal-3-deficient mice [13], the novel observation that the absence of endogenous Gal-1 increased susceptibility to* H. capsulatum* accompanied by higher fungal loads in the lung and spleen was made. Recently, it was reported that Lgals1^−/−^ mice infected intradermally with* T. cruzi* are resistant to this parasitic infection compared to their WT counterparts and this resistant phenotype could be associated with a dysfunction in the regulatory properties of Gal-1 followed by high production of Th1 proinflammatory cytokines and improvement of Th1 and CD8^+^ T cells responses [25]. However, another report from the same group described that Lgals1^−/−^ mice infected intraperitoneally with* T. cruzi* showed elevated parasitemia, less tissue inflammation, and higher mortality rates as compared to infected WT mice [45]. These authors suggest that this discrepancy could be associated with the presence of different phagocytes at sites of infection and distinct local immune response induced by* T. cruzi*. Based on these reports and the present data, it is suggested that the infection of Lgals1^−/−^ mice, intratracheally, with* H. capsulatum* promotes a unique immunophenotype that suppresses the host response against the fungus. This special immunological scenario is characterized by an imbalanced inflammation associated with high levels of neutrophil infiltration and proinflammatory cytokines in the lungs that causes a strong anti-inflammatory response induced by high levels of PGE_2_ and nitric oxide that could modulate phagocyte and T cell functions.

Based on evidence that Gal-4 and Gal-8 can bind and kill bacteria that express a human blood group B-like antigen and a common mammalian antigen *α*-Gal [36, 46], it is hypothesized that Gal-1 might have the same effect on the yeast form of* H. capsulatum. *However, in contrast to Gal-4 and Gal-8 killing activities toward bacteria, Gal-1 neither bound to nor killed the yeast form (Figure 6). This data suggests that the ability of Gal-1 to contribute to proper control of the fungal infection arises from an indirect contribution, since Gal-1 is clearly involved in the modulation of immune response against* H. capsulatum*.

Next, it was evaluated whether the absence of Gal-1 could interfere with the recruitment of neutrophils to the lungs during the infection, since this lectin could modulate the inflammatory response [24, 47]. It is known that neutrophil migration to sites of infection helps the clearance of pathogens [48]. Human neutrophils are able to impair the growth of* H. capsulatum* yeast form, and this microbiostatic effect is mediated mostly by compounds present in the azurophil granules [49]. Moreover, in experimental histoplasmosis, depletion of GR-1^+^ cells, primarily neutrophils, promotes the increase in fungal load in the lungs and spleens and decreases the survival of animals even in the presence of high levels of TNF-*α* and NO [50]. Previous reports demonstrate that mice genetically deficient in Gal-1 have enhanced neutrophil emigration in response to IL-1*β* compared to their wild-type counterparts [51]. Furthermore, in an animal model of zymosan-induced peritonitis, exogenous Gal-1 was shown to cause decreased production of proinflammatory cytokines and expression of adhesion molecules on the surface of neutrophils, thus diminishing their rates of migration [47]. The present results are consistent with those of others, indicating that* H. capsulatum* promotes intense neutrophil recruitment in the lung of Lgals1^−/−^ mice (Figure 3); however, these phagocytes were not able to clear the fungus in the lung pulmonary parenchyma. Other authors have demonstrated that upregulation of proinflammatory cytokines/chemokines resulted in higher numbers of lung neutrophils and also reduced the capacity of the host defense to eliminate the fungus [9]. Since Gal-1 can modulate the adhesion molecules expression as well as releasing mediators of immune response [22–24, 47], it was evaluated whether the increase of neutrophil infiltration into the lung was associated with exacerbation of cytokines during the inflammatory response against* H. capsulatum *infection. The intense neutrophil accumulation in the lung of Lgals1^−/−^ mice could be explained by high levels of IL-1*α* (Figure 4(c)), since this cytokine is a chemoattractant for neutrophils [52, 53]. Moreover, the presence of high number of neutrophils may be a major source of IL-12 detected in the lung from infected-Lgals1^−/−^ mice, as neutrophils have been reported to produce IL-12 [54]. Curiously, inhibition of dectin-1 expression, a host receptor for fungal beta-glucan, reduces the severity of fungus infection and its effect was associated with decrease of proinflammatory cytokines, including IL-12, and neutrophil infiltration [55]. Furthermore, it is known that proinflammatory cytokines, including IL-12 [32, 34, 56, 57], are essential for host defense against* H. capsulatum*. Conversely, on the present model, the increase of IL-12 did not promote fungal clearance in the lungs of Lgals1^−/−^ mice. Based on these results, it may be hypothesized that the excessive production of IL-12p40 and IL-1*α* in Lgals1^−/−^-infected mice is deleterious to the animals. Interestingly, Lgals1^−/−^ mice are more resistant to* Trypanosoma cruzi* infection than wild-type mice and this phenotype is associated with upregulation of IFN-*γ* and no significant production of IL17A [25]. However, the HSV-1 infection in Lgals1^−/−^ mice promotes a severe disease, compare to wild-type, that is correlated with the elevated number of neutrophil infiltrations and IFN-*γ*-producing CD4 T cells and no significant change of IL-17-producing T cell in the ocular [58]. Then, considering that (i) immunoregulatory properties of Gal-1 are associated with regulation of T_H_ 1 and T_H_ 17 responses [59], (ii) IL-12 and IL-23 share p40 subunit [60], and (iii) IL-17/IL-23-axis cytokines participate in immune response against* H. capsulatum *infection [33], further investigation should be done in order to elucidate the impact of IL17/IL23 in experimental histoplasmosis in the absence of endogenous Gal-1. In addition to cytokine production, it was analyzed whether microbicidal factors, such as NO, could be modulated by the deficiency of Gal-1, which could underlie the suppression of host defense against* H. capsulatum*. It was found that the deficiency of Gal-1 promotes the increase of NO concentration in the lung of infected mice when compared with infected WT mice. These results are in concordance with other studies that show that Gal-1 negatively modulates the NO production by activating macrophage or microglia-like cells [23, 61] and activated microglia from Lgals1^−/−^ mice produce high concentration of NO [62]. Moreover, the high levels of NO produced (Figure 4(d)) in the lung have no microbicidal effect on* H. capsulatum, *since lungs from Gal-1* Lgals1* ^−/−^ mice had higher CFU (Figure 2). Thus, NO appears to be important for the host defense against primary infection by* H. capsulatum* [11]; nonetheless, the overproduction of NO has also been shown to suppress phagocytic activities of macrophage in* H. capsulatum* infection and inhibit the CD4 T cells proliferation response to* T. cruzi *infection [12, 34, 50].

Alveolar macrophages are the first line of host defense in the lung against respiratory pathogens, and this phagocyte is an important source of lipid mediators, such as PGE_2_ in infected lung [63]. PGE2 has an important role in suppression of host defense involved modulation of alveolar macrophages functions in different pulmonary infection models, such as* Streptococcus pneumoniae *[64],* Klebsiella pneumoniae* [65],* Pseudomonas aeruginosa* [66], and recently* H. capsulatum* [8]. Lung and macrophages from Lgals1^−/−^-infected mice produced higher levels of PGE_2_ when compared to WT mice. Then, it is hypothesized that high levels of NO and PGE_2_ in the lungs of Lgals1^−/−^-infected mice inhibit the effector functions of macrophages and neutrophils against* H. capsulatum*. Whether the absence of endogenous Gal-1 can inhibit the effector functions of neutrophils against* H. capsulatum* remains unknown, though.

PGE_2_ is able to inhibit IL-12 production by macrophage and dendritic cells [67], although lung parenchyma from infected Lgals1^−/−^ mice contained higher levels of IL-12 than those from infected WT mice even in the presence of high levels of PGE_2_. This finding is in agreement with other studies reporting that the inhibition of prostaglandin has no effects on the production of IL-12 in* H. capsulatum*-infected mice [8]. In addition, the present data is similar to others, demonstrating that the immunoregulatory effects of Gal-1 (endogenous or exogenous) are associated with suppression of Th1 cytokines, including the negative modulation of IL-12 production by activated macrophage or tolerogenic dendritic cells [19, 68–71].

Because of the low yield of murine alveolar macrophages, peritoneal macrophages from Lgals1^−/−^ mice were used to examine the ability of endogenous Gal-1 to modulate the expression of mRNA* Ptges2* and PGE_2_ after* H. capsulatum *infection (Figure 5(b)). The high fungus burden in lungs and spleen in infected Lgals1^−/−^ mice could be associated with the downregulation effects of PGE_2_ in antimicrobial functions of phagocytes [8, 72].

Exaggerated inflammatory response could be responsible for higher production of PGE_2_ in lungs of* H. capsulatum*-infected Lgals1^−/−^ mice that inhibited fungal clearance, since the PGE_2_ biosynthesis is increased under inflammatory conditions, and this prostanoid has been described to impair phagocytosis and kill by alveolar macrophages [73]. In addition, the effector functions of phagocytes from Lgals1^−/−^ could be altered, since Gal-1 is a multifunctional molecule with intra- and extracellular effects [28, 29]. This immune suppressive effect is in line with current results, demonstrating the positive impact on mRNA* Ptgs2* expression and PGE_2_ secretion of the Gal-1 deficiency in macrophages from Lgals1^−/−^ mice after fungal infection. This data is in agreement with the results described by Rabinovich and colleagues, since this lectin can reduce arachidonic acid release and PGE_2_ secretion from activated macrophage [27]. Besides that, celecoxib treatment, a selective cyclooxygenase 2 inhibitor, improved the immune response against* H. capsulatum *infection through the inhibition of prostaglandin production [8]. Curiously, celecoxib induces expression of Gal-1 in activated macrophage and Gal-1 could be involved in the anti-inflammatory mechanisms of this drug [74]. Furthermore, Gal-1 inhibited the expression of activating transcription factor 3, a negative regulator of mRNA* Ptgs2* in macrophage [75]. Despite that, further investigations are needed to elucidate the mechanism by which Gal-1 inhibits* Ptgs2* expression. It has now been shown that PGE_2_ is a DAMP (damage-associated molecular patterns) and is induced and released by dying cells, which leads to suppressed expression of genes associated with inflammation and thereby limits immunostimulatory activities [76]. Also, PGE_2_ is downregulated in human systemic inflammatory diseases and mice with reduced PGE_2_ exhibit systemic inflammation [77]. In summary, the present results demonstrate that the endogenous Gal-1 plays an important role in host defense against* Histoplasma capsulatum* modulating of PGE_2_, IL-12, and NO production, as well as pulmonary neutrophil accumulation. Future studies are needed to better understand the cellular and molecular mechanisms in which endogenous galectin-1 could participate in host defense against fungus infection.

## 4. Materials and Methods

### 4.1. Animals

Six-to-eight-week-old wild-type (WT) male mice and mice genetically deficient in Gal-1 (Lgals1^−/−^), both in a C57BL/6J background, were housed and bred at the animal facility of the School of Pharmaceutical Sciences of Ribeirão Preto (University of São Paulo, Brazil). Wild-type mice were originally purchased from The Jackson Laboratory (Bar Harbor, ME, USA) and Lgals1^−/−^ mice were provided by Dr. Richard D. Cummings (Department of Surgery, Beth Israel Deaconess Medical Center and Harvard Medical School, Boston, MA, USA). The experimental protocol was approved and conducted in accordance with guidelines of the Institutional Animal Care Committee. To check the depletion of Lgals1, Gal-1 expression (mRNA and protein) analysis on WT and Lgals1^−/−^ cells were performed as previously described [21] using conventional RT-PCR and western blot, respectively (data not shown). We used C57BL/6J mice as wild-type counterparts in our experiments.

### 4.2. *H. capsulatum* Strain and Infection of Mice

*H. capsulatum* strain was isolated from a patient at the Clinical Hospital, School of Medicine of Ribeirão Preto, University of São Paulo, and the characterization and preparation of* H. capsulatum* yeast cells were performed as previously described [9, 78, 79]. The yeast cultures were used at ≥90% viability according to fluorescein diacetate (Sigma-Aldrich, St. Louis, MO) and ethidium bromide (Sigma-Aldrich) staining [80]. Mice were given intratracheally (i.t.) dispersion containing 100 *μ*L phosphate buffered saline (PBS, vehicle control) or a sublethal dose in PBS (5 × 10^5^ yeasts/animal). The appropriate inoculum size was chosen based on procedure described by Sá-Nunes and colleagues [78]. On day 15 after infection, both uninfected and infected mice were euthanized in a CO_2_ chamber, and lungs and spleens were collected for analyses.

### 4.3. Fungal Load and Histopathology

*H. capsulatum*-infected mice were euthanized on day 15 after infection and tissue samples were harvested. Lung sections (5 *μ*M) were embedded in paraffin blocks and stained with Grocott's methanamine silver (GMS) and quantification of yeasts was expressed as yeast/mm^2^ (original magnification: 400x). Also, fungal burden was determined from homogenized lung and spleen (Mixer Homogenizer; Labortechnik, Staufen, Germany) as previously described [7, 9]. Serial dilutions of these tissue homogenates were plated onto BHI blood agar and incubated at 37°C for 21 days. The results were expressed as mean colony-forming units (CFU) per gram of lung ± SEM (CFU/g) or CFU per whole spleen ± SEM (CFU/spleen). Lungs were collected, fixed in 10% formaldehyde, and embedded in paraffin blocks. For neutrophils analyses, lung sections (5 *μ*m) were stained with hematoxylin and eosin (H&E) and the cells were quantified in the ocular lens containing 10 × 10 graticules (0.0624 mm^2^ each in magnifications: 400x). The results are expressed as neutrophils/mm^2^.

### 4.4. Measurement of Cytokines, PGE_2_, and Nitric Oxide

Lungs were collected 15 days after infection, weighed and homogenized (Mixer Homogenizer; Labortechnik, Staufen, Germany) in 2 mL of RPMI1640 (Sigma) and the supernatants were stored at −70°C until being assayed. Commercially available ELISA antibodies were used to measure TNF-*α*, IL-1*α*, IL-12p40, IL-10, IL-4, and IL-6 (BD OptEIA ELISA sets; BD Pharmingen) according to the instructions of the manufacturer. PGE_2_ from lung homogenate and from* in vitro* assay (*in vitro* assay is described below) were purified by Sep-Pak C18 cartridges according to the manufacturer's instructions (Waters Corp., Milford, MA). Quantification of PGE_2_ was assessed also by ELISA (Cayman Chemical, Ann Arbor, MI) and the results for cytokines and PGE_2_ are expressed in ng/mL. The sensitivity of the assay was <10 pg/mL. Nitrite (NO_2_^−^) concentrations (*μ*M) in lung homogenates was measured by Griess reaction using a standard curve with serial dilutions of NaNO_2_ (Sigma-Aldrich). Griess reagent was used in order to measure NO levels indirectly from nitrite as described previously [10].

### 4.5. Gene Expression by Real-Time Polymerase Chain Reaction (qRT-PCR)

#### 4.5.1. *In Vitro* Assay

WT or Lgals1^−/−^ peritoneal macrophages (5 × 10^5^ cells/well) were incubated with* H. capsulatum* (MOI 1 : 1) during 2 and 24 hours. PGE_2_ was assessed in the supernatants 24 hours after infection and expression of mRNA was performed in plated macrophages 2 and 24 hours after* H. capsulatum* exposure.

#### 4.5.2. Gene Expression

Total mRNA was isolated using the RNeasy Mini kit (Qiagen Inc., Valencia, CA), according to the manufacturer's instructions. cDNA (complementary DNA) was synthesized from 600 ng of total RNA using random primers (High Capacity cDNA Reverse Transcription Kit, Applied Biosystems, Temecula, CA). Aliquots of 2 *μ*L of the total cDNA were amplified by qRT-PCR (StepOne Plus, Applied Biosystems, Singapore) using the primers (IDT®, Integrated DNA Technologies, California, USA) for* Ptges2* (the gene encoding prostaglandin E synthase 2, Mm.PT. 58.7480753) and probe (TaqMan® Gene Expression Assay, Applied Biosystems, Foster City, USA).* Actb* (Mm00607939) was used as reference gene. Amplification was performed in duplicate under the following conditions: denaturation at 95°C for 10 min, followed by 40 cycles of 95°C for 15 s and 60°C for 1 minute. Relative quantification was performed using the ΔΔCt method and plotted as Fold Increase or Fold Regulation by Log_2_.

### 4.6. Human Recombinant Galectin-1 (hrGal-1) Purification

hrGal-1 was prepared as previously described [26, 81]. Briefly, purified hrGal-1 was treated with 100 mM iodoacetamide (Sigma-Aldrich) in 100 mM lactose/PBS overnight at 4°C [82]. To ensure that hrGal-1 samples were endotoxin-free, Detoxi-Gel Endotoxin removing gel (Pierce Biotechnology, Rockford, IL) was used and hrGal-1 activity was assessed by haemagglutination (data not shown).

### 4.7. Binding by Flow Cytometry and Resazurin Cell Viability Assays

To measure the capacity of Gal-1 to bind on yeast form of* H. capsulatum*, 1 *μ*M and 4 *μ*M biotinylated-hrGal-1 were incubated for 1 hour at 4°C, in presence or absence of 20 mM lactose or sucrose (Sigma-Aldrich). After washing, yeasts were incubated with streptavidin-FITC (Jackson IR) for 30 minutes at 4°C, washed, and formalin-fixed (1% in PBS). Labeled cells were acquired on a FACS Canto (Becton Dickinson, Mountain View, CA, USA) and analyzed in the DIVA software (Becton Dickinson). As a control, we used HL-60 cells obtained from the American Type Culture Collection (ATCC, Manassas, VA) and maintained in RPMI medium supplemented with 10% fetal bovine serum. To test* H. capsulatum* viability in a presence of Gal-1, we incubated,* in vitro*, several hrGal-1 concentrations (10, 4, 2.4, 1, and 0.5 *μ*M) with 1 × 10^6^ yeast cells during 24 and 48 h. The relative fluorescent units (RFU) using a plate reader were detected (560–590 nm) in order to analyze the number of yeast cells metabolically active using the dye resazurin reagent (Sigma-Aldrich).

### 4.8. Statistical Analysis

The data are presented as the mean ± SEM. Comparisons were performed using an ANOVA followed by a Bonferroni posttest by the Prism 4.0 statistical program (GraphPad Software, San Diego, CA). Survival analyses were performed using the Mantel-Cox log-rank (*χ* ^2^ “chi-squared”) test. Differences in survival were analyzed by the log-rank test. Values of *p* < 0.05 were considered statistically significant.

## Abbreviations

Gal-1: Galectin-1
Lgals1^−/−^: Galectin-1 deficient mice
WT: Wild-type mice
Th1 and Th2: Helper T cell responses
IFN: Interferon
IL: Interleukin
TNF-*α*: Tumor necrosis factor-*α*
GM-CSF: Granulocyte macrophage colony-stimulating
PGE_2_: Prostaglandin E2
Ptgs2: Prostaglandin E synthase 2
NO_2_^−^: Nitrite
NO: Nitric oxide
HIV: Human Immunodeficiency Virus-1
HTLV-1: Human T Lymphotropic Virus-1
BALF: Bronchoalveolar Lavage Fluid.
